# Changes over time in the "healthy soldier effect"

**DOI:** 10.1186/1478-7954-9-7

**Published:** 2011-03-14

**Authors:** Michael Waller, Annabel CL McGuire

**Affiliations:** 1 University of Queensland, The Centre for Military and Veterans' Health, Mayne Medical School, 288 Herston Road, Herston, Queensland, 4006, Australia

## Abstract

**Background:**

Death rates in military populations outside of combat are often lower than those in the general population. This study considers how this "healthy soldier effect" changes over time.

**Methods:**

Standardized mortality ratios were used to compare changes in death rates relative to the Australian population in two large studies of Australian servicemen of the Korean War (n = 17,381) and the Vietnam War era (n = 83,908).

**Results:**

The healthy soldier effect was most consistently observed in deaths from circulatory diseases. A large deficit in these deaths in the initial follow-up period (10-20 years) was observed before rates tended to rise to the level seen in the general population. There was no healthy soldier effect in deaths from external causes in enlisted personnel, and these death rates were significantly higher than expected in the initial follow-up period among Korean War veterans and regular Army veterans of the Vietnam War. Those selected for national service during the Vietnam War exhibited the strongest healthy soldier effect of all cohorts assessed.

**Conclusions:**

Patterns of the healthy soldier effect over time varied markedly by study cohort and by cause of death studied. In a number of analyses, the healthy soldier effect was still apparent after more than 30 years of follow-up.

## Introduction

Workers are known to have lower mortality rates than the general population. Good health is necessary to secure and maintain employment, whereas the general population includes sick and disabled people who may be at greater risk of mortality. This observation is known as the "healthy worker effect" and has been frequently reported in occupational epidemiology [1-5].

A similar observation in military populations has been termed the "healthy soldier effect" [6-8]. Kang and Bullman proposed that military personnel would have lower mortality than the general population because of initial physical screens, a requirement to maintain a certain standard of physical well-being, and better access to medical care during military service [6]. This healthy soldier effect may result in an underestimation of the effect of an exposure in studies that use the general population as the comparison group.

McLaughlin et al. conducted a systematic review of literature and compared mortality rates of military personnel with the general population [9]. The results indicated that estimates of the healthy soldier effect were heterogeneous and likely to be strongly dependent on factors such as the type of deployment being studied, the era of the deployment, and differences in the follow-up period between studies.

McMichael et al. observed that the healthy worker effect was likely to be larger at ages closest to recruitment years as employers actively reject unhealthy job applicants [10]. Further, in an analysis that considered 10 occupational cohorts, Monson observed a consistent pattern of the healthy worker effect decreasing with increased follow-up until the standardized mortality ratio (SMR) reached a plateau [5].

In the review by McLaughlin et al., the study that showed the greatest reduction in mortality relative to the general population was the study of Persian Gulf War veterans that followed a young cohort of military personnel for 2.4 years [6]. However, there was no consistent pattern of the healthy soldier effect decreasing with time. Indeed, studies of Korean War technicians [11] and naval personnel [12] with approximately 40 years of follow-up still showed very large mortality reductions.

The first study of the mortality rates of Australian veterans of the Vietnam War was undertaken in the 1980 s [13], and more recently, the mortality of Australians deployed to Korea has been assessed [14]. In order to further understand how the healthy soldier effect changes over time, we used data from the Australian Government Department of Veterans' Affairs (DVA) mortality studies of Australian veterans of the Vietnam and Korean wars to study the temporal pattern of the healthy soldier effect in detail [14-16].

## Methods

### Korean War veterans

The Korean War veteran cohort for this study consisted of 17,381 Australian men who deployed to the Korean War. Mortality records from the Australian Institute of Health and Welfare (AIHW) were held for the years 1950-2000. The Korean War veterans were analyzed as one cohort.

### Vietnam War veterans

Australian personnel deployed to the Vietnam War included national service Army personnel as well as members of the regular Army, Navy, and Air Force. The national service scheme implemented by the Australian government during the Vietnam War required men aged 20 who were normally residents of Australia to register for national service. A total of 804,286 registered for national service, and 237,048 were selected by ballot. After exclusions on medical, psychological, and educational grounds as well as other deferments and exemptions, 63,735 were conscripted for national service [16].

For the Vietnam War study, mortality records were available from 1963-2001. The people in this study were split into the following groups for analysis: Vietnam War regular Army veterans (21,840 men); Vietnam War regular Navy and Air Force veterans (18,100 men); and Vietnam War national service Army veterans (19,239 men). A group of 24,729 men who were conscripted into the Army and completed national service in Australia only are included as a Vietnam War national service comparison group.

### Health outcomes

The primary outcome in this study was all-cause mortality. Deaths from cancer, diseases of the circulatory system, and external causes were also considered separately. Within these categories, mortality from lung cancer, colorectal cancer, ischemic heart disease, stroke, motor vehicle accidents, and suicide are also presented. Vietnam and Korean War veterans were followed up from the date when they returned from deployment until their date of death or the end of follow-up (whichever was earliest). Follow-up was over the lifetime of the individual after deployment, and not just for the period they served in the Australian Defence Force. Since the national service comparison group had no date of returning to Australia, follow-up began on the day that they turned 22 years old.

### Statistical analysis

SMRs were used to compare rates in the veteran populations to Australian population norms. The SMR is the ratio of the observed and the expected numbers of deaths.

The vital status of participants in these cohorts had been determined by computerized matching of veterans' records with information in large national databases, such as the AIHW National Death Index (NDI), the electoral roll, DVA databases, and other registries. The primary analyses included those in the at-risk population whose vital status was unknown, assuming that they were still alive and residing in Australia. However, the data were also analyzed excluding those whose vital status was unknown from the at-risk population to assess the effect of this assumption.

The numbers of deaths in the Australian population were obtained from published reports [17]. The expected number of deaths was calculated by multiplying the number of person-years in each five-year age group (up to 85+) for each calendar year by the mortality rate in males for that age group and year in the Australian population. Confidence intervals for the SMRs were calculated using Poisson regression.

The SMRs were tabulated by follow-up interval to show how the death rates differed from those in the Australian population in each time period. Graphs are also provided as an additional file (Additional file 1) to show how the SMRs changed by cumulative year of follow-up (that is, the overall SMR from the start of follow-up to the year in question). All data were analyzed using STATA 10.0 [18].

### Ethics approvals

Ethics approval for this study was received from the Department of Veterans' Affairs Ethics Committee, the Australian Institute of Health and Welfare Ethics Committee, and the University of Queensland Behavioural & Social Sciences Ethical Review Committee.

## Results

### Demographic characteristics

The demographic characteristics of the Korean War veteran study personnel and the Vietnam War veteran study cohorts on return from their respective deployments are shown in Table 1. The average length of follow-up per person for the Korean War and Vietnam War veterans was 41 years (SD 10 years) and 32 years (SD 5 years), respectively.

**Table 1:** Characteristics of the Korean War veterans, Vietnam War veterans, and national service nonveterans (comparisons)

	Korean War Veterans	Vietnam War Regular Army Veterans	Vietnam War Regular Navy and Air force Veterans	Vietnam War National Service Army Veterans	Total Vietnam War Veterans	Vietnam War National Service Comparisons
***Characteristics***		**Frequency (%)**	**Frequency (%)**	**Frequency (%)**	**Frequency (%)**	**Frequency (%)**

Age*						
*15-19*	627 (4%)	1725 (8)	4940 (27)	53 (0.3)	6718 (11)	
*20-24*	8375 (48%)	9643 (44)	6585 (36)	18823 (98)	35051 (59)	24729 (100)
*25-29*	5470 (31%)	4216 (19)	2928 (16)	363 (2)	7507 (13)	
*30-34*	1663 (10%)	2596 (12)	1447 (8)		4043 (7)	
*35+*	1246 (7%)	3660 (17)	2200 (12)		5869 (7)	
						
*Service*						
*Navy*	5742 (33%)		13534 (75)		13534 (23)	
*Army*	10482 (60%)	21840 (100)		19239 (100)	41079 (69)	24729 (100)
*Air Force*	1157 (7%)		4566 (25)		4566 (8)	
						
*Total*	17381	21840	18100	19239	59179	24729

*Age on return to Australia

### Korean War veterans

Rates of all-cause mortality in the Korean War veterans for the follow-up period 1950-2000 were higher than those observed in the general population (overall SMR 1.11; 95% confidence interval [CI]: 1.08, 1.13).

In the initial 20 years of follow-up, death rates from all-causes mortality in the Korean War veterans were similar to those in the general population (Table 2). However, after 25 years of follow-up, all-cause mortality rates rose relative to those of the general population, peaking 30 to 39 years after follow-up began (SMR 1.19; 95% CI: 1.15, 1.24).

**Table 2:** Standardised Mortality Ratios (SMR) by period of follow-up for all Korean War veterans

*Years of follow-up*	*0-9*			*10-19*			*20-29*			*30-39*			*40+*		
	O*	E^†^	SMR 95% CI	O	E	SMR 95% CI	O	E	SMR 95% CI	O	E	SMR 95% CI	O	E	SMR 95% CI
***All deaths***	383	360.1	1.06 (0.96, 1.18)	674	680.7	0.99 (0.92, 1.07)	1435	1343.3	1.07 (1.01, 1.12)	2590	2168.4	1.19 (1.15, 1.24)	2432	2240.0	1.09 (1.04, 1.13)
***Cancers***	31	42.0	0.74 (0.52, 1.05)	109	116.4	0.94 (0.78, 1.13)	381	339.1	1.12 (1.02, 1.24)	985	745.1	1.32 (1.24, 1.41)	964	825.8	1.17 (1.10, 1.24)
*Lung cancer*	2	4.5	0.44 (0.11, 1.77)	24	27.4	0.88 (0.59, 1.31)	152	107.4	1.42 (1.21, 1.66)	317	230.2	1.38 (1.23, 1.54)	303	223.2	1.36 (1.21, 1.52)
*Colorectal cancer*	3	4.5	0.66 (0.21, 2.05)	14	14.8	0.95 (0.56, 1.60)	40	45.7	0.87 (0.64, 1.19)	129	100.5	1.28 (1.08, 1.53)	111	106.4	1.04 (0.87, 1.26)
***Circulatory diseases***	62	73.4	0.85 (0.66, 1.09)	256	277.8	0.92 (0.82, 1.04)	669	637.7	1.05 (0.97, 1.13)	1015	942.1	1.08 (1.01, 1.15)	890	880.2	1.01 (0.95, 1.08)
*Ischemic heart disease*	27	35.5	0.76 (0.52, 1.10)	180	195.5	0.92 (0.80, 1.07)	471	470.3	1.00 (0.92, 1.10)	725	666.4	1.09 (1.01, 1.17)	547	565.9	0.97 (0.89, 1.05)
*Stroke*	15	14.3	1.05 (0.63, 1.74)	31	41.3	0.75 (0.53, 1.07)	88	87.7	1.00 (0.81, 1.24)	145	130.1	1.11 (0.95, 1.31)	168	153.3	1.10 (0.94, 1.27)
***External causes***	256	176.9	1.45 (1.28, 1.64)	215	166.4	1.29 (1.13, 1.48)	179	135.2	1.32 (1.14, 1.53)	109	96.7	1.13 (0.93, 1.36)	54	57.7	0.94 (0.72, 1.22)
*Motor vehicle accidents*	87	80.5	1.08 (0.88, 1.33)	51	60.8	0.84 (0.64, 1.10)	39	43.6	0.89 (0.65, 1.22)	28	24.6	1.14 (0.79, 1.65)	7	12.2	0.57 (0.27, 1.20)
*Suicides*	38	33.4	1.14 (0.83, 1.56)	48	46.5	1.03 (0.78, 1.37)	53	39.3	1.35 (1.03, 1.77)	42	33.4	1.26 (0.93, 1.70)	28	18.5	1.51 (1.05, 2.19)

*Observed deaths

^†^Expected deaths

There was a statistically significant excess of deaths from external causes in the first 10 years of follow-up (SMR 1.45; 95% CI: 1.28, 1.64), and these external causes death rates remained significantly higher than those of the general population over the first 30 years of follow-up, after which point they returned to the population level. The number of motor vehicle accidents was close to the population level in each decade of follow-up; however, the number of suicides was higher than in the general population for the follow-up intervals 20-29 years and 40+ years.

Cancer and circulatory disease mortality rates were initially lower than expected before gradually rising after 20 years of follow-up. Rates of cancer (all sites) and lung cancer mortality were significantly higher than in the general population after 20 years of follow-up. The cumulative mortality from cancers over the entire follow-up period was 19% higher than expected in the general population, whereas the observed rate of deaths from circulatory diseases was approximately equal to the population level over the same period.

### Vietnam War regular Army veterans

The all-cause mortality rate among the Vietnam War regular Army veterans was very similar to that of the Australian population across the entire follow-up period (Table 3). When mortality rates in the first 10 years of follow-up were investigated by cause of death, there was a clear excess of deaths due to external causes (SMR 1.27; 95% CI: 1.13, 1.44) due in part to deaths from motor vehicle accidents (SMR 1.40; 95% CI: 1.19, 1.65). In contrast, there was a significant deficit in deaths from diseases of the circulatory system (SMR 0.66; 95% CI: 0.53, 0.82) in the same time period. After the first 10 years, death rates from external causes dropped to the level observed in the Australian population, while death rates from diseases of the circulatory system gradually increased.

**Table 3:** Standardised Mortality Ratios (SMR) by period of follow-up for all Vietnam War regular Army veterans

*Follow-up*	*0-9 years*			*10-19 years*			*20-29 years*			*30+ years*		
	O*	E^†^	SMR 95% CI	O	E	SMR 95% CI	O	E	SMR 95% CI	O	E	SMR 95% CI
***All deaths***	464	485.6	0.96 (0.87, 1.05)	702	749.4	0.94 (0.87, 1.01)	1256	1309.9	0.96 (0.91, 1.01)	571	561.6	1.02 (0.94, 1.10)
***Cancers***	71	73.2	0.97 (0.77, 1.22)	208	201.6	1.03 (0.90, 1.18)	491	476.1	1.03 (0.94, 1.13)	261	226.3	1.15 (1.02, 1.30)
*Lung cancer*	10	14.0	0.71 (0.38, 1.33)	52	53.3	0.98 (0.74, 1.28)	135	124.8	1.08 (0.91, 1.28)	73	56.8	1.28 (1.02, 1.62)
*Colorectal cancer*	8	8.6	0.93 (0.47, 1.87)	27	27.1	1.00 (0.68, 1.45)	60	65.6	0.91 (0.71, 1.18)	28	30.6	0.91 (0.63, 1.32)
***Circulatory diseases***	83	125.6	0.66 (0.53, 0.82)	253	275.1	0.92 (0.81, 1.04)	408	464.5	0.88 (0.80, 0.97)	176	188.9	0.93 (0.80, 1.08)
*Ischemic heart disease*	57	85.5	0.67 (0.51, 0.86)	209	198.5	1.05 (0.92, 1.21)	302	322.0	0.94 (0.84, 1.05)	120	125.2	0.96 (0.80, 1.15)
*Stroke*	14	19.2	0.73 (0.43, 1.23)	21	35.2	0.60 (0.39, 0.92)	52	63.4	0.82 (0.62, 1.08)	28	30.0	0.93 (0.64, 1.35)
***External causes***	268	210.3	1.27 (1.13, 1.44)	147	145.9	1.01 (0.86, 1.18)	127	116.4	1.09 (0.92, 1.30)	28	29.8	0.94 (0.65, 1.36)
*Motor vehicle accidents*	145	103.5	1.40 (1.19, 1.65)	47	46.8	1.00 (0.75, 1.34)	29	25.9	1.12 (0.78, 1.61)	4	6.1	0.66 (0.25, 1.74)
*Suicides*	39	44.8	0.87 (0.64, 1.19)	49	48.1	1.02 (0.77, 1.35)	55	47.9	1.15 (0.88, 1.49)	11	11.6	0.95 (0.52, 1.71)

*Observed deaths

^†^Expected deaths

Death rates from cancer in this group were at a similar level to the general population in the first 30 years of follow-up. However, after more than 30 years, observated rates of cancer mortality (SMR 1.15; 95% CI: 1.02, 1.30) and lung cancer mortality (SMR 1.28; 95% CI: 1.02, 1.62) were significantly higher than the population level.

### Vietnam War regular Navy and Air Force veterans

In the first 20 years of follow-up, regular Navy and Air Force Vietnam veterans had all-cause death rates that were significantly lower than those observed in the general population (Table 4). After 20 years of follow-up, all-cause death rates were equivalent to those observed in the Australian population.

**Table 4:** Standardised Mortality Ratios (SMR) by period of follow-up for Vietnam War regular Navy and Air Force veterans

*Follow-up*	*0-9 years*			*10-19 years*			*20-29 years*			*30+ years*		
	O*	E^†^	SMR 95% CI	O	E	SMR 95% CI	O	E	SMR 95% CI	O	E	SMR 95% CI
***All deaths***	291	364.8	0.80 (0.71, 0.89)	456	521.5	0.87 (0.80, 0.96)	922	893.9	1.03 (0.97, 1.10)	452	451.1	1.00 (0.91, 1.10)
***Cancers***	44	49.0	0.90 (0.67, 1.21)	129	131.1	0.98 (0.83, 1.17)	385	314.5	1.22 (1.11, 1.35)	185	181.1	1.02 (0.88, 1.18)
*Lung cancer*	9	8.3	1.09 (0.57, 2.09)	44	32.7	1.35 (1.00, 1.81)	107	80.3	1.33 (1.10, 1.71)	51	45.0	1.13 (0.86, 1.49)
*Colorectal cancer*	8	5.3	1.51 (0.75, 3.01)	13	17.2	0.76 (0.44, 1.30)	54	43.1	1.25 (0.96, 1.63)	24	24.7	0.97 (0.65, 1.45)
***Circulatory diseases***	44	77.9	0.57 (0.42, 0.76)	144	177.3	0.81 (0.69, 0.96)	298	307.6	0.97 (0.86, 1.09)	144	149.1	0.97 (0.82, 1.14)
*Ischemic heart disease*	23	51.2	0.45 (0.30, 0.68)	107	126.6	0.85 (0.70, 1.02)	210	214.5	0.98 (0.86, 1.12)	102	100.2	1.02 (0.84, 1.24)
*Stroke*	16	12.4	1.29 (0.79, 2.10)	18	23.0	0.78 (0.49, 1.24)	41	41.0	1.00 (0.74, 1.36)	19	22.5	0.84 (0.54, 1.32)
***External causes***	180	183.5	0.98 (0.85, 1.13)	130	124.3	1.05 (0.88, 1.24)	102	98.2	1.04 (0.86, 1.26)	28	29.1	0.96 (0.66, 1.39)
*Motor vehicle accidents*	92	97.1	0.95 (0.77, 1.16)	44	41.9	1.05 (0.78, 1.41)	14	22.3	0.63 (0.37, 1.06)	6	5.9	1.02 (0.46, 2.27)
*Suicides*	19	35.6	0.53 (0.34, 0.84)	38	40.0	0.95 (0.69, 1.31)	53	40.6	1.30 (1.00, 1.71)	11	11.8	0.93 (0.52, 1.68)

*Observed deaths

^†^Expected deaths

Mortality rates from external causes in this group were not significantly different from those in the Australian population at any time over the follow-up period, although the number of suicides in the first decade of follow-up was lower than the population level (SMR 0.53; 95% CI: 0.34, 0.84). For deaths from circulatory disease, a strong healthy soldier effect in the first 20 years was followed by a period where mortality rates were close to those observed in the general population.

Cancer mortality rates were similar to those of the general population for the first 20 years of follow-up, rising to a significant excess between 20 and 30 years (SMR 1.22; 95% CI: 1.11, 1.35), before falling back to general population levels after 30 years of follow-up. In this cohort, there was an excess of lung cancer deaths relative to the general population for the second and third decades of follow-up.

### Vietnam national service Army veterans

The Vietnam national service Army veterans had all-cause mortality rates that were significantly lower than those observed in the general population for the duration of follow-up (Table 5).

**Table 5:** Standardised Mortality Ratios (SMR) by period of follow-up for Vietnam War national service Army veterans

*Follow-up*	*0-9 years*			*10-19 years*			*20-29 years*			*30+ years*		
	O*	E^†^	SMR 95% CI	O	E	SMR 95% CI	O	E	SMR 95% CI	O	E	SMR 95% CI
***All deaths***	206	274.0	0.75 (0.66, 0.86)	236	298.1	0.79 (0.70, 0.90)	436	540.9	0.81 (0.73, 0.88)	174	184.9	0.94 (0.81, 1.09)
***Cancers***	22	26.4	0.83 (0.55, 1.27)	56	56.0	1.00 (0.77, 1.30)	146	171.1	0.85 (0.73, 1.00)	74	75.4	0.98 (0.78, 1.23)
*Lung cancer*	1	0.9	1.12 (0.16, 7.97)	9	6.3	1.42 (0.74, 2.74)	35	34.6	1.01 (0.73, 1.41)	22	16.6	1.33 (0.87, 2.02)
*Colorectal cancer*	2	1.5	1.31 (0.33, 5.26)	4	6.3	0.63 (0.24, 1.69)	14	22.9	0.61 (0.36, 1.03)	10	9.9	1.01 (0.54, 1.87)
***Circulatory diseases***	15	20.0	0.75 (0.45, 1.24)	38	63.3	0.60 (0.44, 0.82)	116	152.6	0.76 (0.63, 0.91)	44	53.0	0.83 (0.62, 1.12)
*Ischemic heart disease*	6	7.0	0.86 (0.39, 1.92)	30	41.3	0.73 (0.51, 1.04)	94	107.2	0.88 (0.72, 1.07)	33	37.6	0.88 (0.62, 1.23)
*Stroke*	3	4.7	0.64 (0.21, 1.98)	3	8.8	0.34 (0.11, 1.06)	6	18.6	0.32 (0.14, 0.72)	4	6.0	0.66 (0.25, 1.77)
***External causes***	161	192.4	0.84 (0.72, 0.98)	114	125.3	0.91 (0.76, 1.09)	89	104.5	0.85 (0.69, 1.05)	21	21.6	0.97 (0.63, 1.49)
*Motor vehicle accidents*	81	105.9	0.77 (0.62, 0.95)	34	41.2	0.83 (0.59, 0.87)	15	22.4	0.67 (0.40, 1.11)	2	4.0	0.50 (0.12, 1.98)
*Suicides*	27	35.8	0.75 (0.52, 1.10)	41	42.0	0.98 (0.72, 1.33)	51	45.2	1.13 (0.86, 1.48)	11	9.3	1.18 (0.65, 2.13)

*Observed deaths

^†^Expected deaths

This group had a significant deficit of deaths from external causes for the first 10 years of follow-up. A significant deficit in the first decade was also observed in deaths from motor vehicle accidents and suicides. Deaths from circulatory diseases were consistently lower than in the general population, an effect that was most clearly observed between 10 and 20 years of follow-up (SMR 0.60; 95% CI: 0.44, 0.82). There was also a significant deficit of deaths from strokes between 20 and 29 years of follow-up. No statistically significant differences were observed between this group and the Australian population in death rates from all-sites cancer, lung cancer, or colorectal cancer across the follow-up period.

### Vietnam national service Army nonveterans (comparisons)

The Vietnam War national service Army nonveterans exhibited the strongest healthy soldier effect of all cohorts studied. For all-cause deaths, there was a clear deficit of mortality across the entire follow-up period (Table 6).

**Table 6:** Standardised Mortality Ratios (SMR) by period of follow-up for Vietnam War national service Army nonveterans (comparisons)

*Follow-up*	*0-9 years*			*10-19 years*			*20-29 years*			*30+ years*		
	O*	E^†^	SMR 95% CI	O	E	SMR 95% CI	O	E	SMR 95% CI	O	E	SMR 95% CI
***All deaths***	204	351.9	0.58 (0.51, 0.67)	240	385.1	0.62 (0.55, 0.71)	484	688.6	0.70 (0.64, 0.77)	161	238.0	0.68 (0.58, 0.79)
***Cancers***	28	33.6	0.83 (0.58, 1.21)	49	72.3	0.68 (0.51, 0.90)	171	217.2	0.79 (0.68, 0.91)	72	97.5	0.74 (0.59, 0.93)
*Lung cancer*	1	1.2	0.87 (0.12, 6.15)	3	8.1	0.37 (0.12, 1.15)	31	43.8	0.71 (0.50, 1.01)	13	21.7	0.60 (0.35, 1.03)
*Colorectal cancer*	2	1.9	1.03 (0.26, 4.10)	6	8.1	0.74 (0.33, 1.64)	28	29.0	0.96 (0.67, 1.40)	10	12.9	0.78 (0.42, 1.44)
***Circulatory diseases***	8	25.7	0.31 (0.16, 0.62)	51	81.1	0.63 (0.48, 0.83)	148	194.1	0.76 (0.65, 0.90)	47	68.3	0.69 (0.52, 0.92)
*Ischemic heart disease*	4	8.9	0.45 (0.17, 1.19)	35	52.8	0.66 (0.48, 0.92)	103	136.2	0.76 (0.62, 0.92)	31	48.5	0.64 (0.45, 0.91)
*Stroke*	0	6.1	-	8	11.3	0.71 (0.35, 1.41)	18	23.8	0.76 (0.48, 1.20)	6	7.7	0.78 (0.35, 1.73)
***External causes***	159	247.6	0.64 (0.55, 0.75)	117	161.8	0.72 (0.60, 0.87)	90	133.8	0.67 (0.55, 0.83)	16	27.3	0.59 (0.36, 0.96)
*Motor vehicle accidents*	71	136.0	0.52 (0.41, 0.66)	33	53.0	0.62 (0.44, 0.88)	17	28.8	0.59 (0.37, 0.95)	4	5.0	0.79 (0.30, 2.12)
*Suicides*	15	46.5	0.32 (0.19, 0.54)	49	54.5	0.90 (0.68, 1.19)	43	57.8	0.74 (0.55, 1.00)	8	11.8	0.68 (0.34, 1.35)

*Observed deaths

^†^Expected deaths

The pattern of change in the SMR due to deaths from external causes mirrored that of all-cause mortality. Although the magnitude of the deficit was smaller, the effect was still statistically significant. The number of motor vehicle accidents was significantly lower than the population level in the first three decades of follow-up, and the number of suicides was lower than the expected level in the first 10 years of the study (SMR 0.32; 95% CI: 0.19, 0.54).

The national service Army nonveterans were the only group studied to show a consistent deficit of deaths from all-sites cancer mortality. Death rates due to diseases of the circulatory system were also lower than those of the general population. This deficit of circulatory disease deaths, due in part to lower mortality from ischemic heart disease, gradually declined over time but was still clear at the end of follow-up in 2001 (SMR 0.69; 95% CI: 0.61, 0.78).

### Adjustment for unknown death status

Excluding those whose death status was unknown from the at-risk population resulted in the all-cause SMRs associated with the study of Korean War veterans increasing by between 6% and 12%. The effect of this adjustment in the Vietnam War cohorts was smaller (an increase in SMRs of between 1% and 3%).

## Discussion

The estimates of the healthy soldier effect were markedly different among the cohorts studied in these analyses (Table 7). Among Korean War veterans, there was no evidence of a healthy soldier effect for total deaths. In the Vietnam War veterans, a clear healthy soldier effect for all-cause mortality was observed in the national service groups and among the Navy and Air Force personnel. Such an effect was not observed in the Vietnam War regular Army cohort.

**Table 7:** Summary table of healthy soldier effect in Australian military cohorts

Cause	Significant deficit of deaths relative to the general population			Healthy soldier effect declines with increased follow-up
	**0-9 years**	**10-19 years**	**20-29 years**	

***All causes***	VRNAV, VNSAV, VNSAC	VRNAV, VNSAV, VNSAC	VNSAV, VNSAC	VRNAV, VNSAV, VNSAC
***Cancers***		VNSAC		KV, VRNAV
***Circulatory diseases***	VRAV, VRNAV, VNSAC	VRNAV, VNSAV, VNSAC	VRAV, VNSAV, VNSAC	KV, VRAV, VRNAV, VNSAV, VNSAC
***External causes***	VNSAV, VNSAC	VNSAC	VNSAC	

KV = Korean War veterans

VRAV = Vietnam War regular Army veterans

VRNAV = Vietnam War regular Navy and Air Force veterans

VNSAV = Vietnam War national service Army veterans

VNSAC = Vietnam War national service Army nonveterans (comparisons)

Temporal trends in the healthy soldier effect varied by cause of death. Where present, the healthy soldier effect was observed most clearly in all-cause death rates and deaths from circulatory diseases. Indeed, a consistent observation was an initial deficit in deaths from circulatory diseases that declined with increasing follow-up. In general, the trends of deaths from ischemic heart disease mirrored those from the circulatory disease category. In each cohort, deaths from stroke were generally similar to the level observed in the general population, although comparisons were often limited by low numbers of observed and expected events in some of the 10-year time periods.

Healthy soldier effects on all-sites cancer mortality, lung, and colorectal cancer mortality were not clearly observed in these analyses. These trends were difficult to assess in the initial follow-up period because most cancer types are more common at older ages. Nevertheless, it has been suggested that a healthy soldier effect on cancer mortality is less likely to be observed in occupational cohorts because the factors that will predict eventual death from cancer cannot be easily detected on entry to the workforce [5].

In each of the enlisted (regular) cohorts, there was an excess of cancer deaths between 20 and 40 years of follow-up. It has been hypothesized that this observed increase in cancer mortality may be due in part to smoking patterns or chemical exposures experienced in the respective deployments to Korea and Vietnam [14,15]. This increase in deaths from cancer was not observed among national service personnel. Given that the national service cohorts were the youngest of those studied, it will be of interest to observe future changes in cancer mortality rates in these groups.

There was no evidence of a healthy soldier effect for deaths due to external causes, except in the Vietnam War national service veterans and nonveterans. In both the Korean War veterans and the Vietnam regular Army veterans, there were clear excesses of deaths due to external causes in the initial period of follow-up that may have cancelled out the expected healthy soldier effect in all-cause mortality in the initial follow-up period in these cohorts. This may indicate that some members of the Defence Force are vulnerable to deaths from external causes in the period after returning from deployment. Common causes within this category were suicides and deaths from motor vehicle accidents. It has been hypothesized that an excess in deaths from external causes may be due in part to risk-takers' attraction to a military career [19]. This may help explain why no excess in deaths from external causes was present in the national service cohorts.

Previous research has shown the duration of the healthy worker effect to vary between five and 25 years [1,5], and after such time, the effect has been shown to plateau but not necessarily at the population level [20]. Circulatory disease death rates in Korean War Army veterans followed this pattern. The Vietnam War regular Army and regular Navy/Air Force groups also exhibited a plateau of circulatory disease deaths below the level in the Australian population occurring between 10 years (regular Army) and 20-25 years (regular Navy and Air Force).

After a strong initial deficit of mortality in the Vietnam War national service Army and nonveteran groups, only a small reduction of the healthy soldier effect was observed. However, these were the youngest of all of the cohorts studied (the majority were under 56 years old at the end of follow-up), and therefore, further follow-up of this group may be necessary to observe the point at which the healthy soldier effect is no longer present.

The reduced mortality in national service veterans was observed in both the group that deployed to Vietnam and the group that did not. The national service program allowed the Australian Army to select from the population more educated and skilled personnel than it had been attracting at that time [16]. In addition, the selection process of national service veterans involved a series of medical, psychological, and educational assessments. Consequently, the criteria used to select these personnel may have resulted in a healthy soldier effect stronger than that observed in the selection of the enlisted groups.

The Korean War veterans' cohort was distinctive in that they had an excess of all-cause mortality at the end of follow-up. The initial excess in all-cause mortality in the Korean veterans had disappeared by 20-25 years of follow-up, after which point all-cause mortality rates increased. At least 30% of the Korean War veterans served in World War II, and involvement in more than one conflict may have contributed to this observed increase in mortality [14]. The increased level of mortality in Korean War veterans relative to the general population may also be due in part to the characteristics of the Australian population used as a comparison group. Immigration rates were relatively high through the 1950 s and 1960 s [21], and these new immigrants would have passed the necessary health screens to enter Australia and been of a similar age to the Korean War veterans. Therefore, the excess of deaths observed in the Korean veterans may have been higher than if the comparison group were required to have been living in Australia at the time of the Korean War.

A question of interest that this study has been unable to answer is how the mortality experience of a military population changes when personnel leave the Defence Force. In a study of Royal Naval personnel, Inskip et al. observed that mortality rates of those who left the military were closer to that of the general population [22]. This finding is intuitive in that personnel may leave the Defence Force because of ill health and then no longer be required to maintain a minimum standard of health in their new employment. An analysis of these temporal trends of current and ex-serving personnel would be valuable to further understand this interaction.

These analyses have highlighted the complexity of the healthy soldier effect. This effect was most clearly observed in circulatory system deaths. The results emphasize that the healthy soldier effect changes over time and varies markedly among different causes of death and different subgroups of the Defence Force. As such, crude adjustments that use a standard correction factor [2] to account for the healthy soldier effect are unlikely to be adequate in studies of military personnel that use the general population as a comparison group, even after accounting for the length of follow-up. Where possible, internal comparison groups (i.e., from a Defence Force population) should be used in studies that aim to assess the effect of a particular exposure on Defence Force members. In some instances, a comparison of Defence Force personnel with an alternative employed group may be of use to ensure that the comparison is between two cohorts healthy enough to maintain employment. Nevertheless, a comparison with the general population may still be of interest to observe which exposure groups' outcomes are closest to those of the general population.

## List of abbreviations and acronyms

AIHW: Australian Institute of Health and Welfare: DVA: Department of Veterans' Affairs: SMR: Standardized mortality ratio

## Competing interests

The authors declare that they have no competing interests.

## Authors' contributions

MW performed the statistical analysis and wrote the manuscript. AM assisted with the management of the project and contributed to the manuscript. All authors have read and approved the final manuscript.

## Supplementary Material

Additional file 1 **Figures 1-20 illustrating the changes in the SMRs by cumulative year of follow-up in each cohort for all-cause mortality, external cause mortality, cancer mortality, and circulatory disease mortality**. Click here for file
