# Supplementary figures and images for: Changes over time in the "healthy soldier effect"
Source: Popul Health Metr. 2011 Mar 14;9:7. doi: 10.1186/1478-7954-9-7 (PMC3062595; doi:10.1186/1478-7954-9-7)

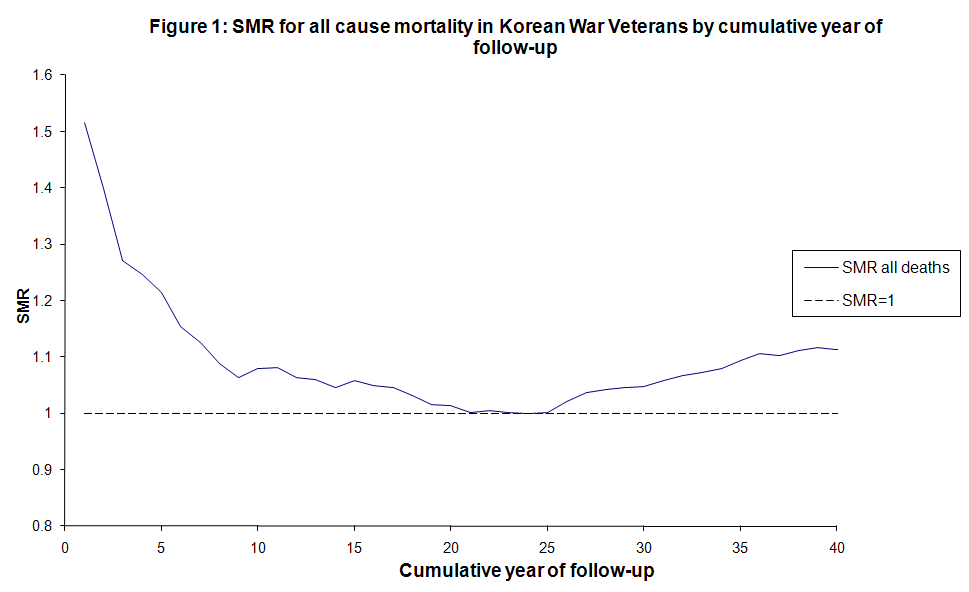

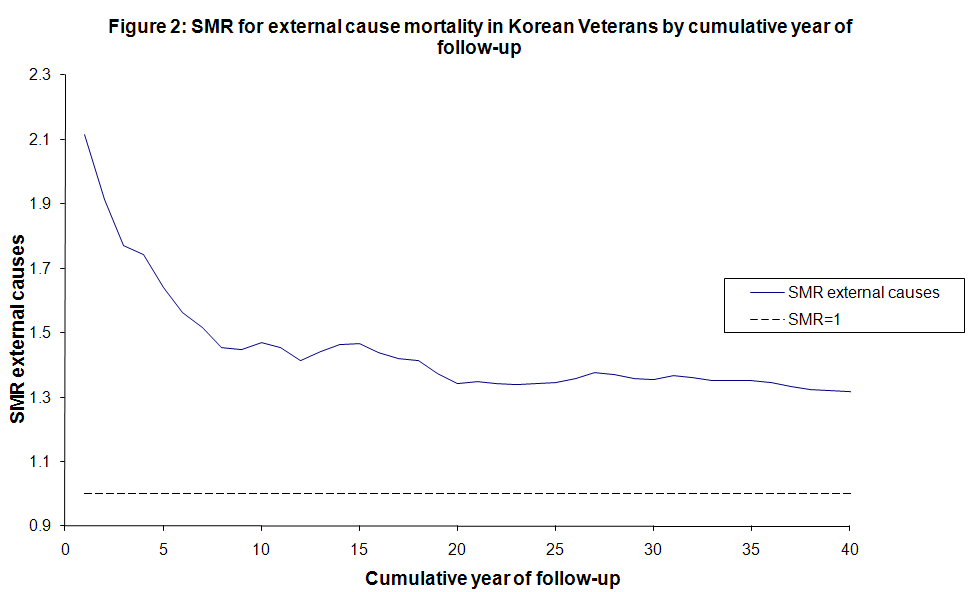

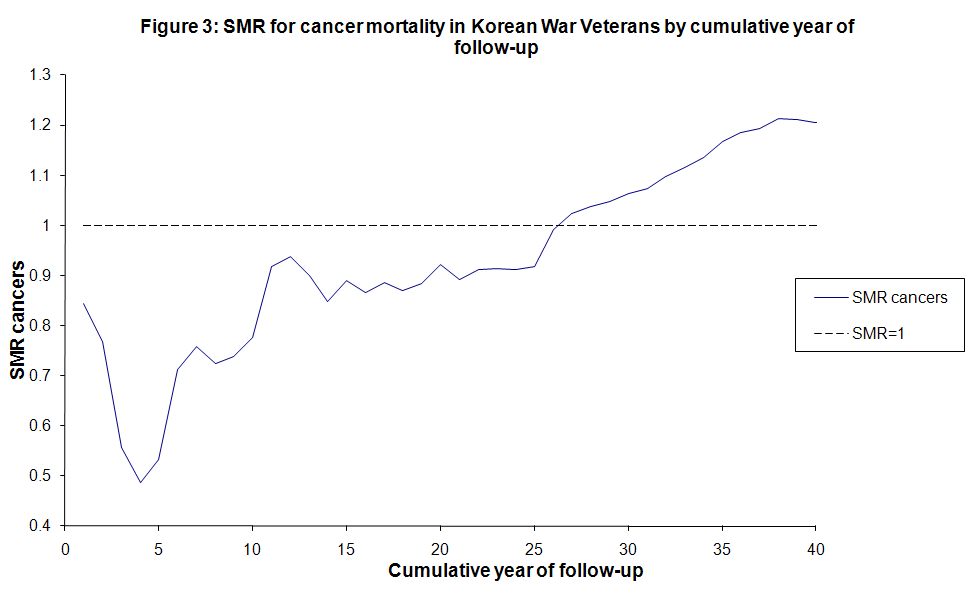

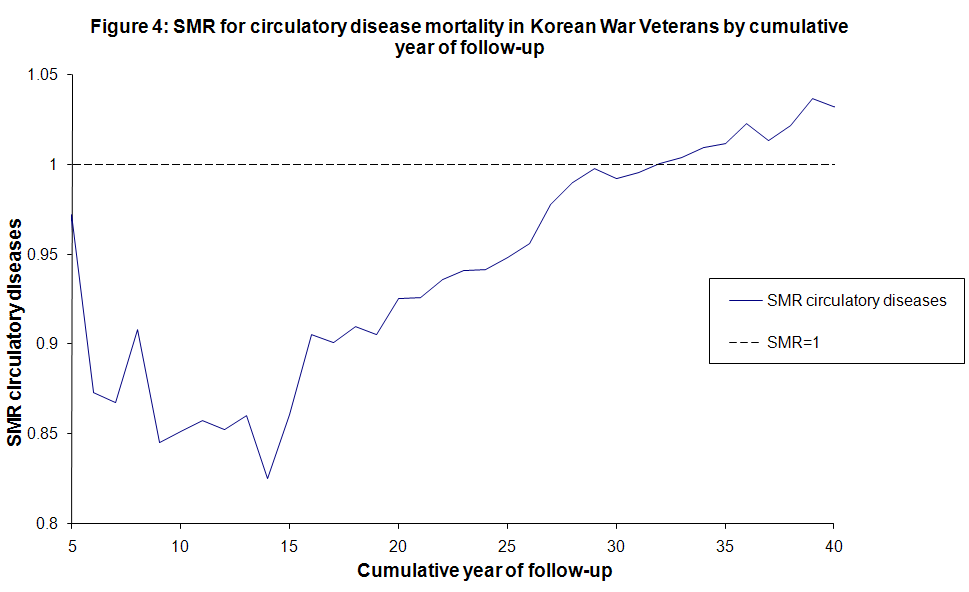

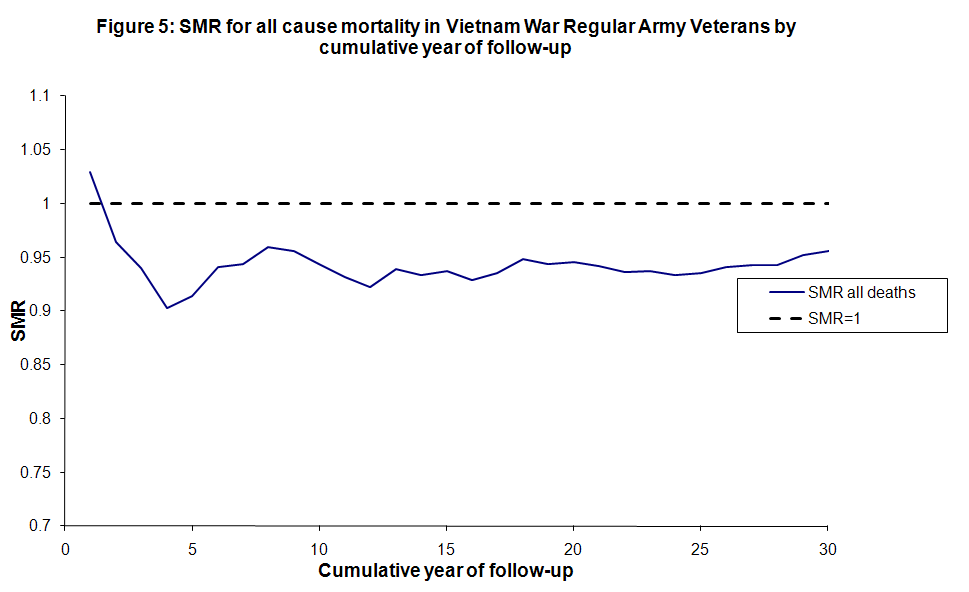

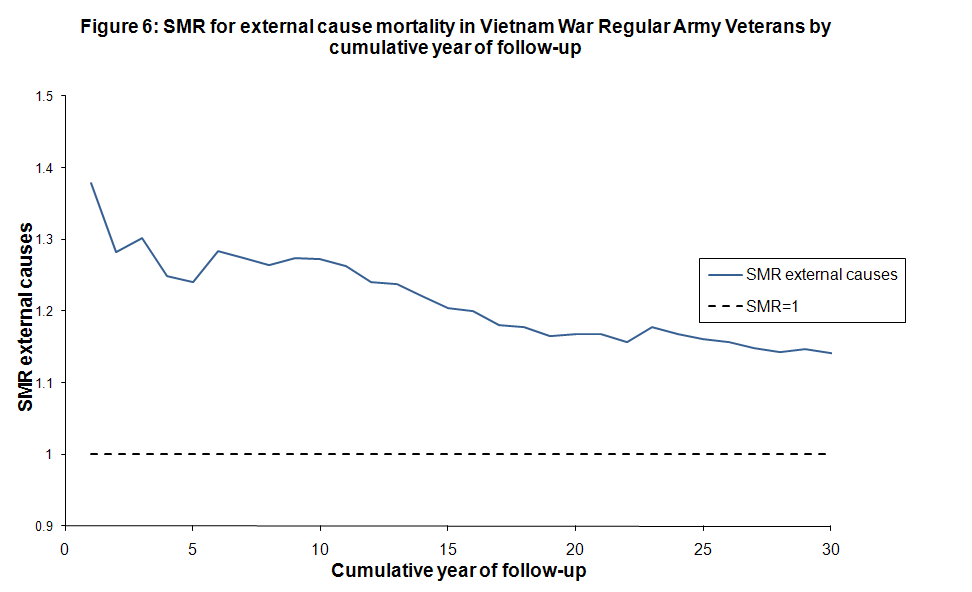

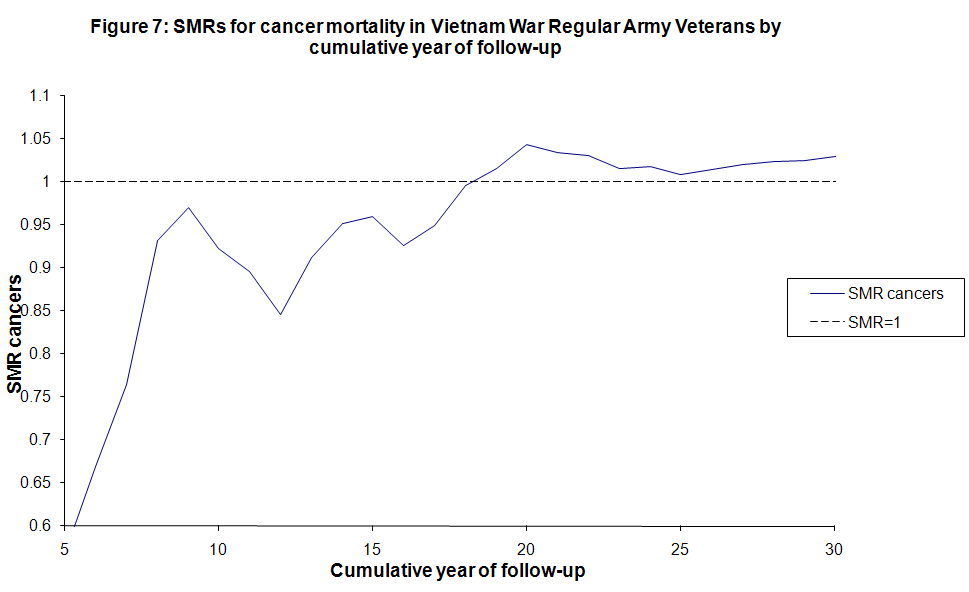

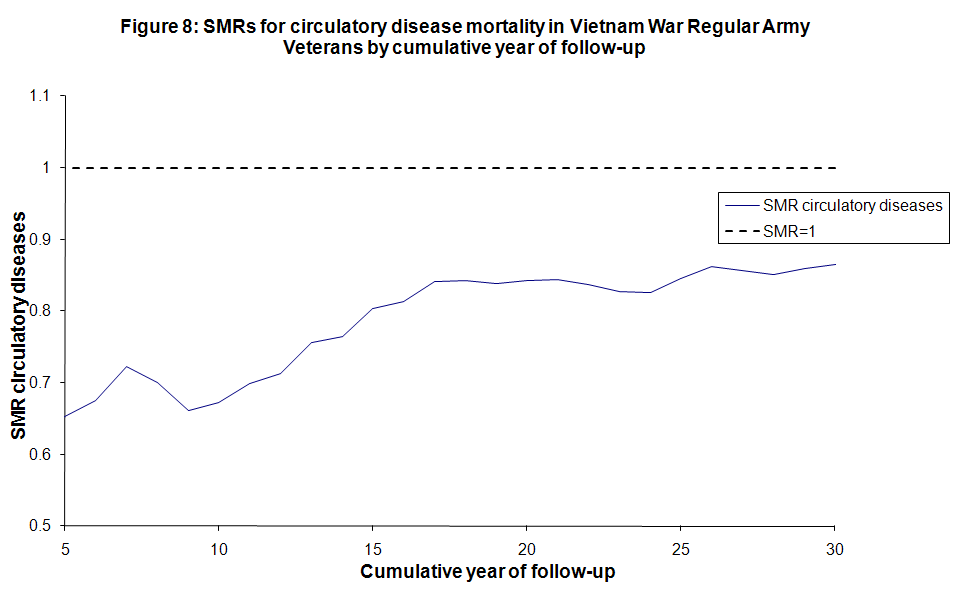


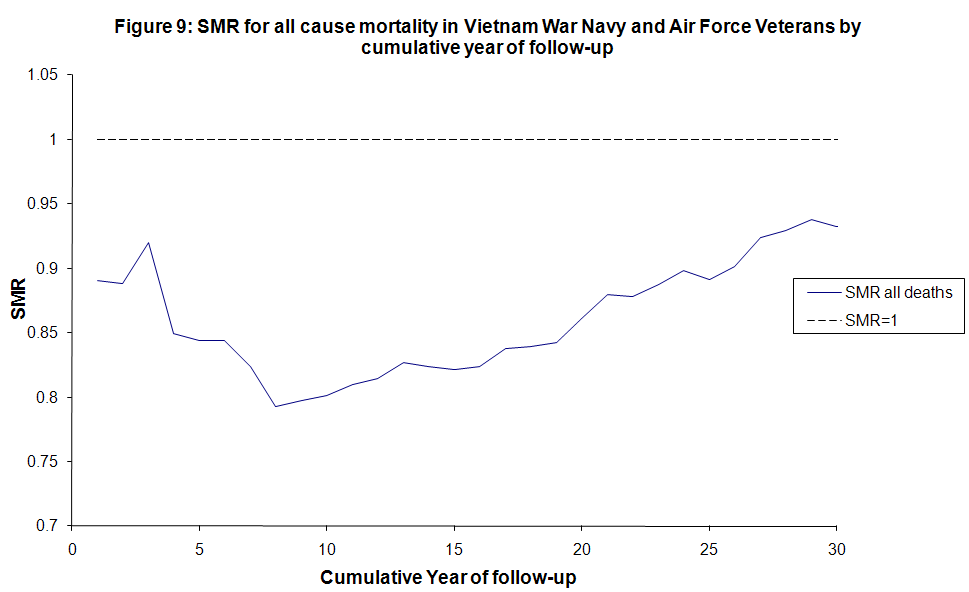

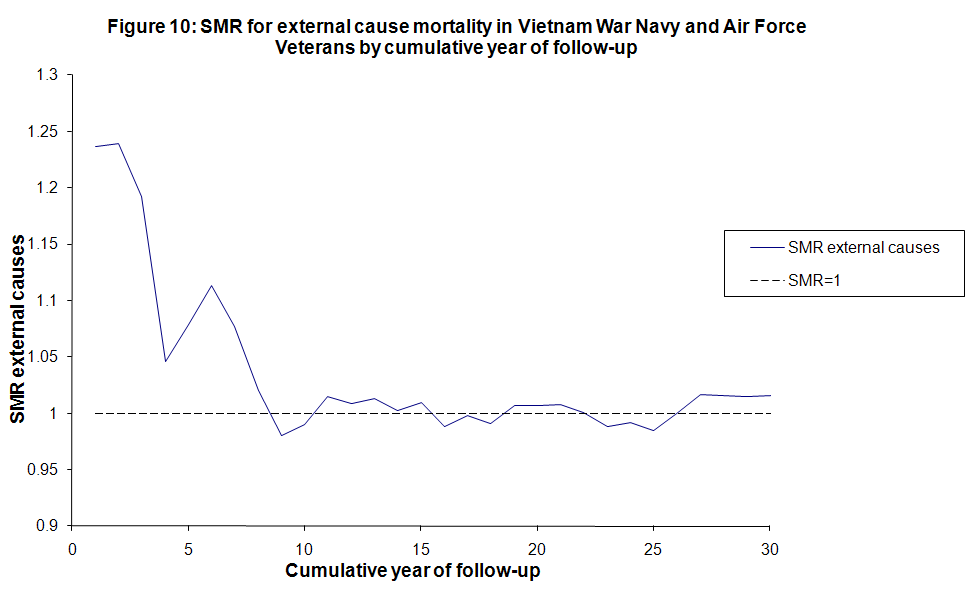

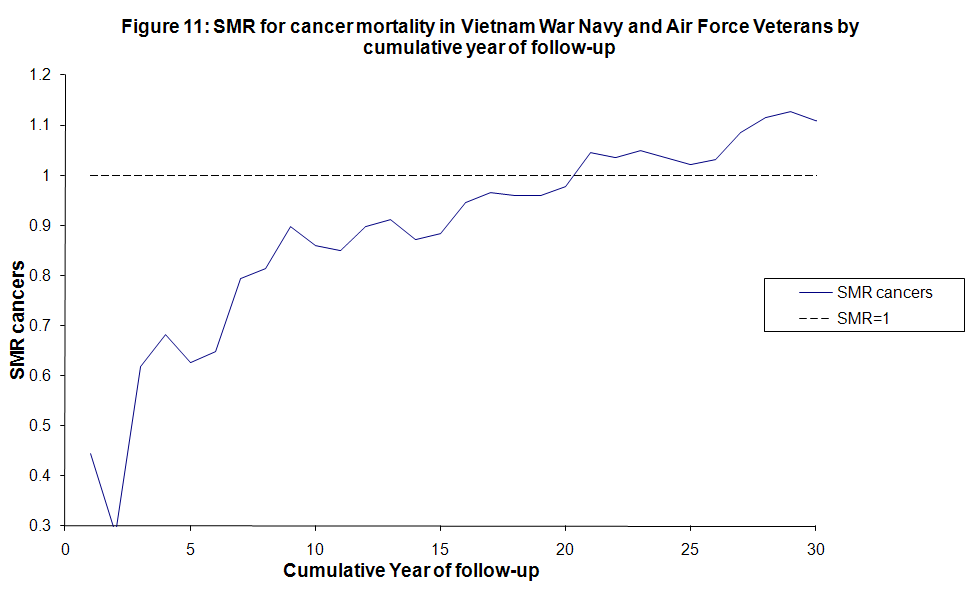

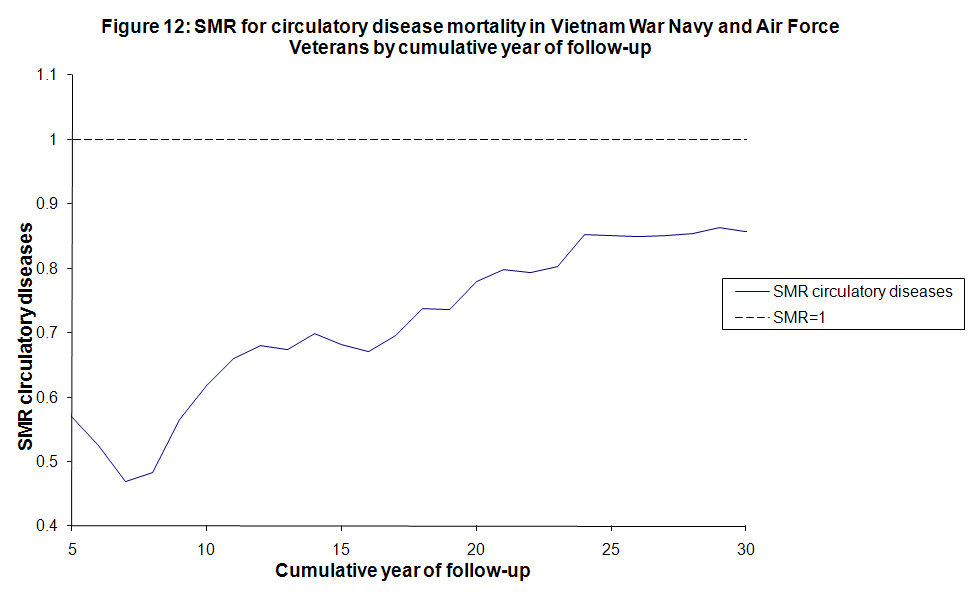

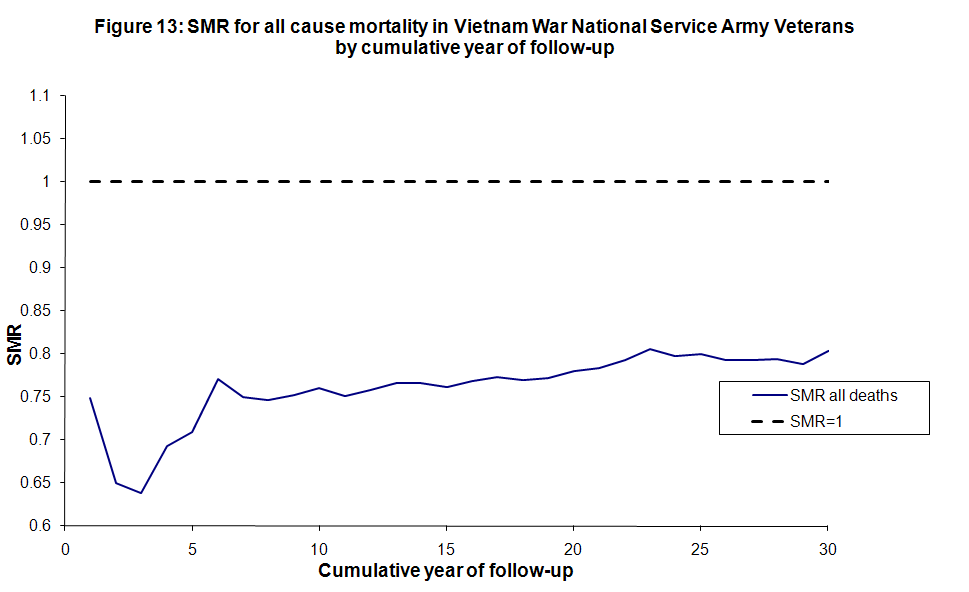

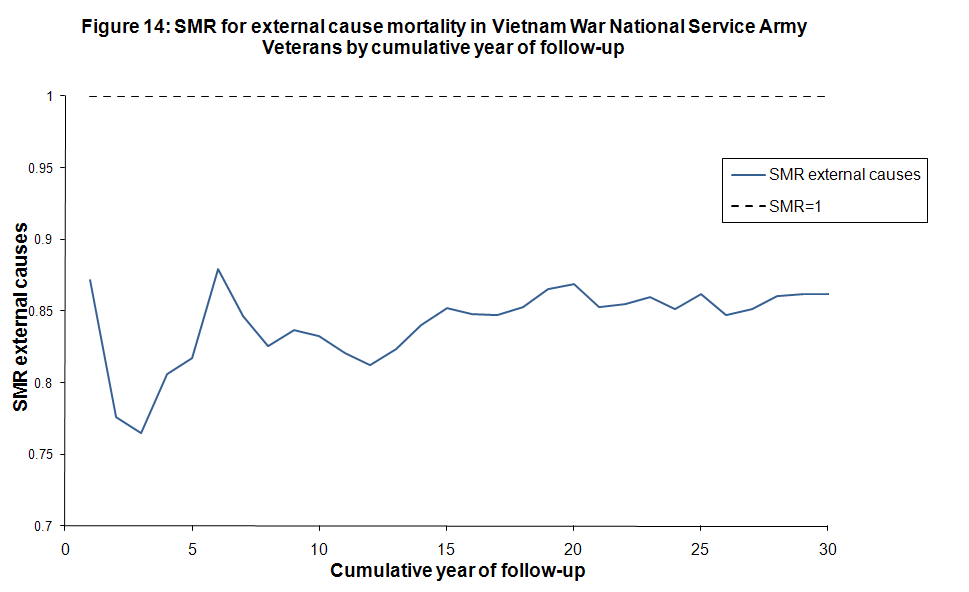

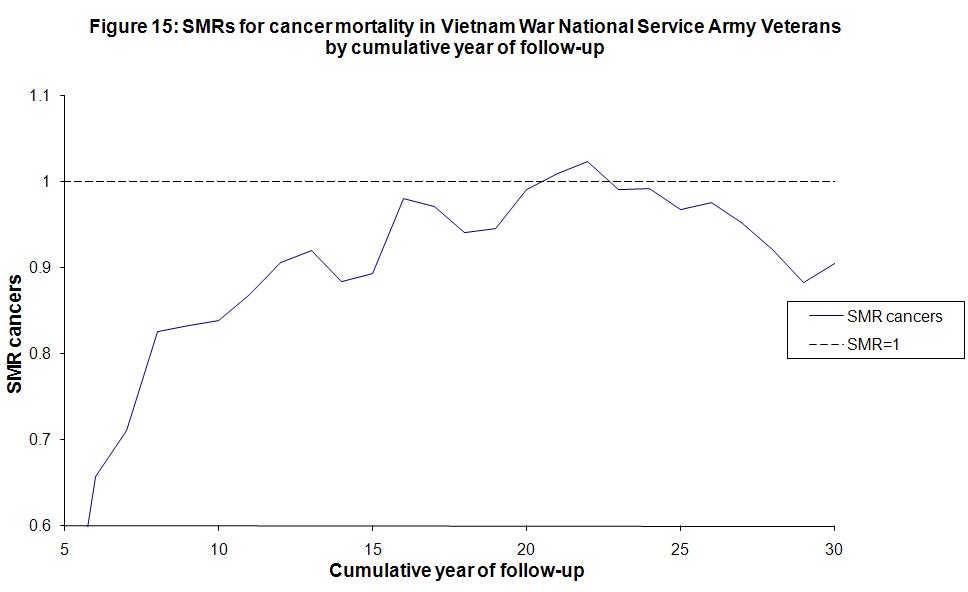

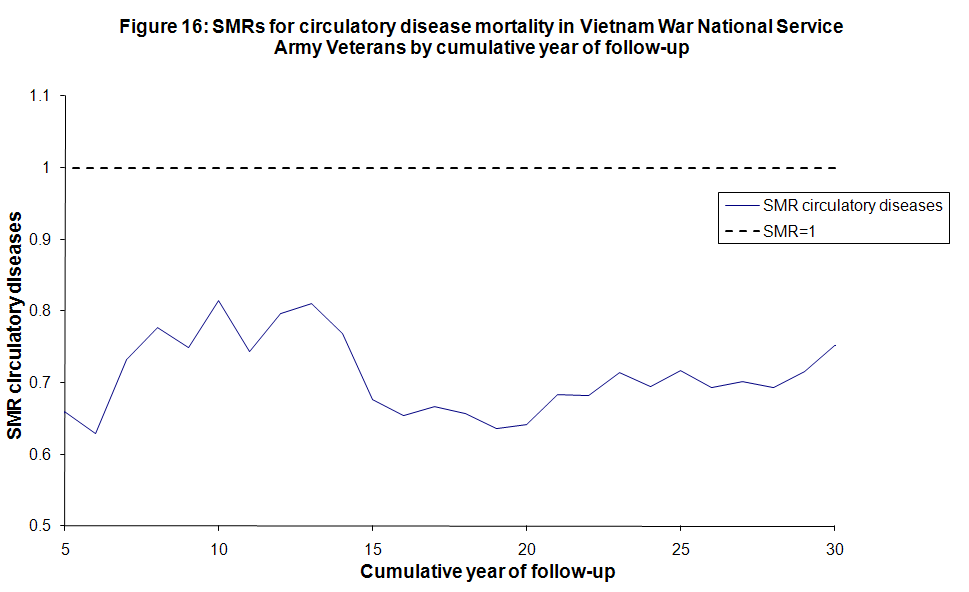

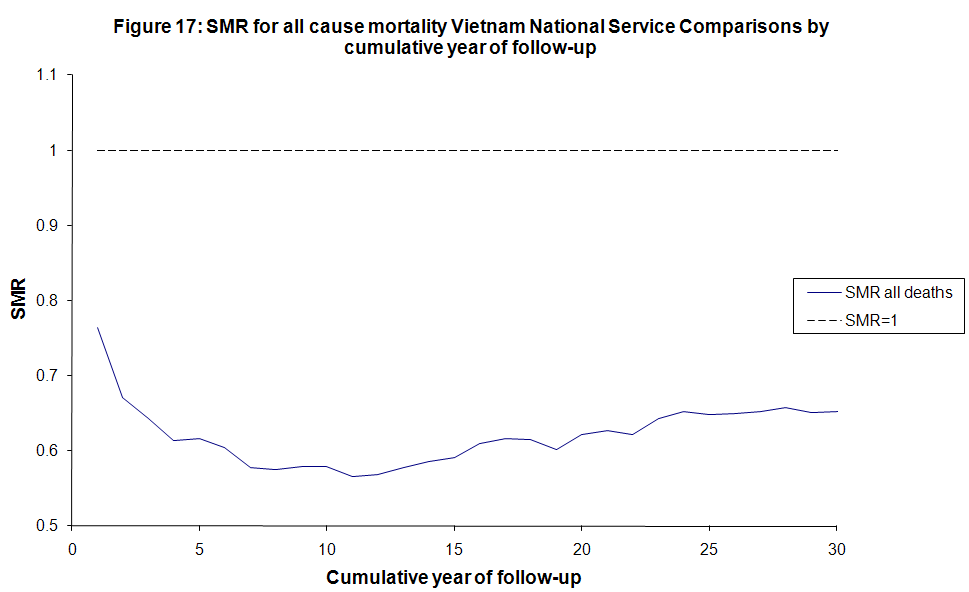

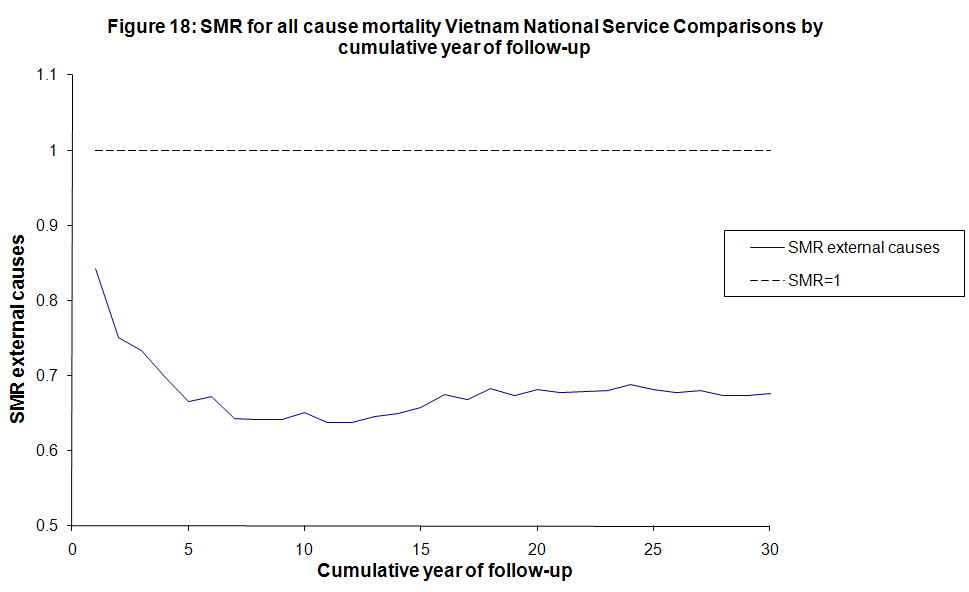

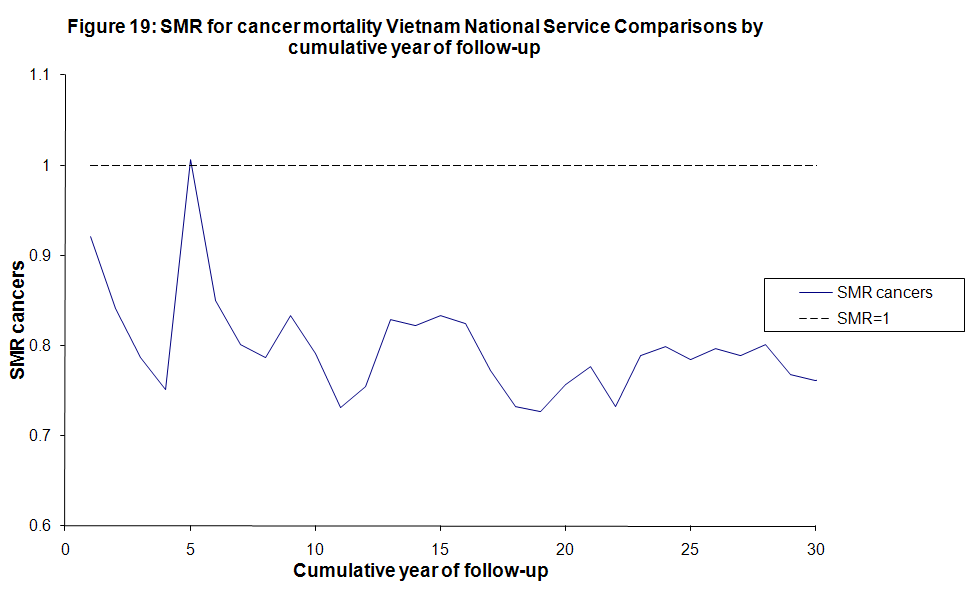

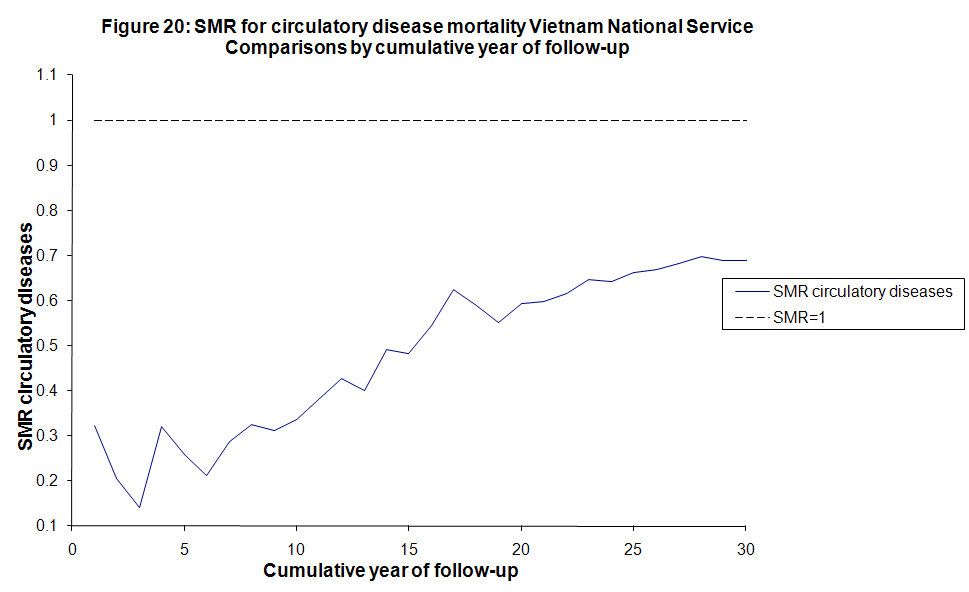

Supplement: Additional file 1 — Figures 1-20 illustrating the changes in the SMRs by cumulative year of follow-up in each cohort for all-cause mortality, external cause mortality, cancer mortality, and circulatory disease mortality. [file 1478-7954-9-7-S1.DOC]
